# Effect of Chemotherapy Cytarabine and Acute Myeloid Leukemia on the Development of Spermatogenesis at the Adult Age of Immature Treated Mice

**DOI:** 10.3390/ijms23074013

**Published:** 2022-04-04

**Authors:** Bara’ah Khaleel, Eitan Lunenfeld, Joseph Kapelushnik, Mahmoud Huleihel

**Affiliations:** 1 The Shraga Segal Department of Microbiology, Immunology, and Genetics, Faculty of Health Sciences, Ben-Gurion University of the Negev, Beer Sheva 8410501, Israel; khaleelb@post.bgu.ac.il; 2 Faculty of Health Sciences, Ben-Gurion University of the Negev, Beer Sheva 8410501, Israel; 3 The Center of Advanced Research and Education in Reproduction (CARER), Faculty of Health Sciences, Ben-Gurion University of the Negev, Beer Sheva 8410501, Israel; lunenfld@bgu.ac.il (E.L.); kapelush@bgu.ac.il (J.K.); 4 Faculty of Medicine, Ariel University, Ariel 4076414, Israel; 5 Department of Pediatric Oncology and Hematology, Soroka Medical Center, Beer-Sheva, and Faculty of Health Sciences, Ben-Gurion University of the Negev, Beer-Sheva 8410501, Israel

**Keywords:** spermatogenesis, acute myeloid leukemia (AML), male infertility, chemotherapy, immature mice, pre-pubertal, testis, apoptosis

## Abstract

Acute myeloid leukemia (AML) accounts for around 20% of diagnosed childhood leukemia. Cytarabine (CYT) is involved in the AML treatment regimen. AML and CYT showed impairment in spermatogenesis in human and rodents in adulthood. We successfully developed an AML disease model in sexually immature mice. Monocytes and granulocytes were examined in all groups: untreated control, AML alone, CYT alone and AML+CYT (in combination). There was a significant increase in the counts of monocytes and granulocytes in the AML-treated immature mice (AML) compared to the control, and AML cells were demonstrated in the blood vessels of the testes. AML alone and CYT alone impaired the development of spermatogenesis at the adult age of the AML-treated immature mice. The damage was clear in the structure/histology of their seminiferous tubules, and an increase in the apoptotic cells of the seminiferous tubules was demonstrated. Our results demonstrated a significant decrease in the meiotic/post-meiotic cells compared to the control. However, CYT alone (but not AML) significantly increased the count of spermatogonial cells (premeiotic cells) that positively stained with SALL4 and PLZF per tubule compared to the control. Furthermore, AML significantly increased the count of proliferating spermatogonial cells that positively stained with PCNA in the seminiferous tubules compared to the control, whereas CYT significantly decreased the count compared to the control. Our result showed that AML and CYT affected the microenvironment/niche of the germ cells. AML significantly decreased the levels growth factors, such as SCF, GDNF and MCSF) compared to control, whereas CYT significantly increased the levels of MCSF and GDNF compared to control. In addition, AML significantly increased the RNA expression levels of testicular IL-6 (a proinflammatory cytokine), whereas CYT significantly decreased testicular IL-6 levels compared to the control group. Furthermore, AML alone and CYT alone significantly decreased RNA expression levels of testicular IL-10 (anti-inflammatory cytokine) compared to the control group. Our results demonstrate that pediatric AML disease with or without CYT treatment impairs spermatogenesis at adult age (the impairment was more pronounced in AML+CYT) compared to control. Thus, we suggest that special care should be considered for children with AML who are treated with a CYT regimen regarding their future fertility at adult age.

## 1. Introduction

Acute myeloid leukemia (AML) is a heterogeneous blood disorder characterized by immature myeloid cell proliferation and bone marrow failure [1]. Although AML may occur at any age, its incidence increases with age [2]. Pediatric AML accounts for 20% of diagnosed childhood leukemia. However, the cure rate is approaching approximately 70% [3]. Epidemiologically, pediatric AML has several possible risk factors, such as infectious, genetic and environmental factors, especially ionizing radiation, which has been significantly linked with AML [2].

A significant reduction in sperm parameters, such as sperm counts, concentration and motility, has been shown in adult leukemia patients compared to healthy donors and other examined adult cancer patients [4,5,6]. Recently, our group established an AML mouse model to evaluate the effect of AML alone and in combination with CYT on the fertility of adult males [7,8]. We showed that AML has an impairment effect on the histology of the testes, spermatogenesis process and sperm parameters, increased spontaneous acrosome reaction, and decreased male fertility capacity and the number of offspring [7,8]. A few reports showed that testicular myeloid sarcoma was reported in pediatric AML patients without showing the histology of the testes [9,10,11,12]. Additional studies that used testicular biopsies from prepubertal cancer patients, including those with AML, were focused on the presence of spermatogonial cells in those biopsies and the negative effect of chemotherapy on those cells or showed histology of some seminiferous tubules (most were normal, and some were abnormal) [13,14,15]. 

The treatment of AML patients under the age of 60 often includes two chemotherapeutic medications: cytarabine (CYT) and anthracycline drugs, such as daunorubicin, idarubicin and/or mitoxantrone [16,17]. In general, chemotherapies cause gonadotoxicity because they target rapidly proliferating cells [18]. The harmful effect of chemotherapies on spermatogenesis depends on several parameters, such as the type of treatment, dosage, initial semen quality, and the regimen of therapy [19]. According to a previous study on rats, injection of CYT (intraperitoneal; i.p) into four-week-old rats showed that CYT impaired spermatogenesis and negatively affected testicular development and function by inducing DNA damage and apoptosis and reducing germ cell proliferation [20]. Furthermore, it decreased sperm count in the treated group compared to the control group. Furthermore, histologically, CYT causes tubular distortion and mild vacuolization in rat testes [20].

Spermatogenesis is a complicated biological process of spermatogonial cell division and maturation into highly specialized haploid spermatozoa [21]. Spermatogonial cells represent the stem cell population of the germ cells, which is located in the basement membrane of the seminiferous epithelium [22]. Spermatogonial cells constitute a heterogeneous population that expresses several markers, such as the transcription factors Sal-like 4 (SALL4) and promyelocytic leukemia zinc finger (PLZF), that could be detected in the undifferentiated type A spermatogonia [23]. The final division produces spermatocytes that undergo the extended prophase of meiosis for around 14 days in mice, followed by the first division to produce secondary spermatocytes (SS) and the second division to produce the haploid spermatid. This is followed by the transformation of round spermatids into elongated (spermiogenesis), highly condensed and mature spermatozoa that are later released into the seminiferous tubule lumen [24]. Both spermatids are expressed activator cAMP-responsive element modulators (CREMs), which are considered meiotic stage markers, whereas the meiotic and post-meiotic stages include elongated spermatid and spermatozoa-expressed ACROSIN [25].

The microenvironment that surrounds the germ cells plays a key role in the regulation of spermatogenesis. Somatic cells (Sertoli cells, Leydig cells and peritubular cells) secret proinflammatory and immunoregulatory cytokines (IL-10 and IL-6) under normal conditions and in response to inflammatory stimuli [26], in addition to growth factors (GDNF, SCF, MCSF and LIF) that regulate the proliferation/differentiation and apoptosis of SSCs [27,28,29,30,31]. Imbalance in testicular cytokines and growth factors may impair normal spermatogenesis [32].

In the present study, we developed an AML-treated immature mouse model and evaluated the direct effect of AML and cytarabine (CYT), alone and in combination, on the development of different stages of spermatogenesis post treatment.

## 2. Results

### 2.1. Development of AML in Immature Mice

**Figure 1:**
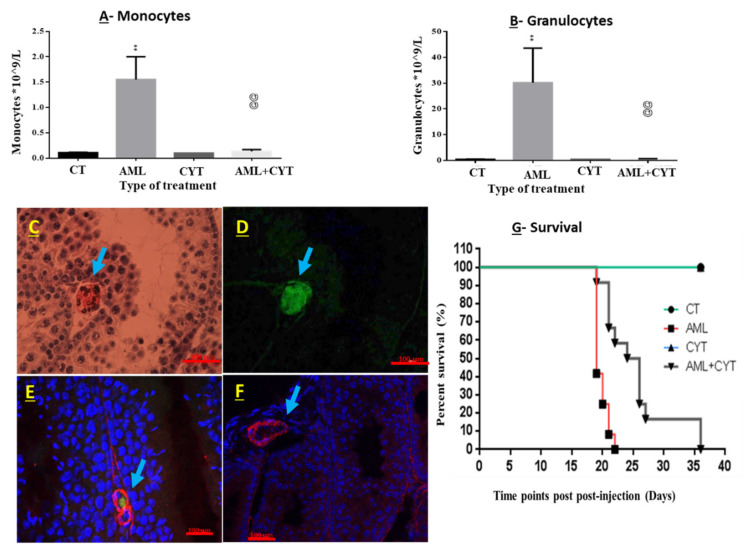
Effect of AML cell line and cytarabine (CYT) on monocytes and granulocytes in the blood vessels, as well as survival of immature treated mice. Two-week-old mice were divided into four groups: untreated (control; CT), injected with AML cells ((i.p.) AML), injected with cytarabine (i.p.) three times and injected with AML cells and cytarabine (AML + CYT). Some of the mice were killed 2 weeks post injection, and the some were kept for survival analysis. Blood was immediately collected from the heart. Monocyte (**A**) and granulocyte (**B**) fractions were then scored. N (number of repeats of all treatments) = 2; n (number of mice examined in all the experiments for each treatment) = 5–9. Fixed testicular tissue was isolated (**C**–**F**) from GFP-AML-injected mice (**C**–**E**) and AML-injected mice without GFP; hematoxylin- and eosin-stained sections (**C**,**D**). H&E-stained sections under green light (**D**). Testicular tissues were immunoassayed with a blood vessel cell marker (a-smooth) (red color) (**E**,**F**). Arrows indicate the location of blood vessel in the testis. Survival of the mice was followed-up within 35 days (**G**). N (number of repeats of all treatments) = 1–3; n (number of mice examined in all the experiments for each treatment) = 11–12. ** (*p* < 0.01) significant relative to control group. @@ (*p* < 0.01) significant in relative to AML group.

Two-week-old mice were injected i.p. with AML cells. Two weeks later, the mice were sacrificed, and blood was immediately collected from the heart (by cardiac puncture) for counting of monocytes and granulocytes. There was a 10-fold increase in monocytes and a 30-fold increase in granulocytes in the AML cell-injected mice compared to uninjected control mice (Figure 1A,B). Additional confirmation for the presence of AML cells in the blood following i.p injection of the AML cells was demonstrated by i.p. injection of AML-GFP cells. Two weeks post injection, mice were sacrificed, and testes were collected, fixed and paraffin-imbedded. Testicular tissue was sectioned and examined for the presence of AML-GFP cells in the testicular blood vessel. Blood vessels in the interstitial compartment of the testis (Figure 1C) and the AML-GFP cells inside the blood vessels were visualized using fluorescence microscopy (Figure 1D; green color) (Figure 1E; green color merge with blue (the color of the nucleus of the cells)). 

### 2.2. Effect of AML and CYT on the Survival of Immature Treated Mice

The survival of the AML- and CYT-treated immature mice was examined for 6 weeks post treatment. Our results show that AML-injected immature mice survived around three weeks post injection, whereas the AML+CYT-injected immature group survived for 36 days post injection (Figure 1G). The CT group and the CYT (alone)-injected group survived for the duration of the experiment (Figure 1G).

### 2.3. Effect of AML and CYT on Testes Weight and the Testicular Histology of Immature Treated Mice

To evaluate the effect of AML and CYT on the testes, we collected testes from all study groups at different time points (1 week–4.5 weeks according to the survival of the mice) post treatment with AML, CYT, AML+CYT or control. 

**Figure 2:**
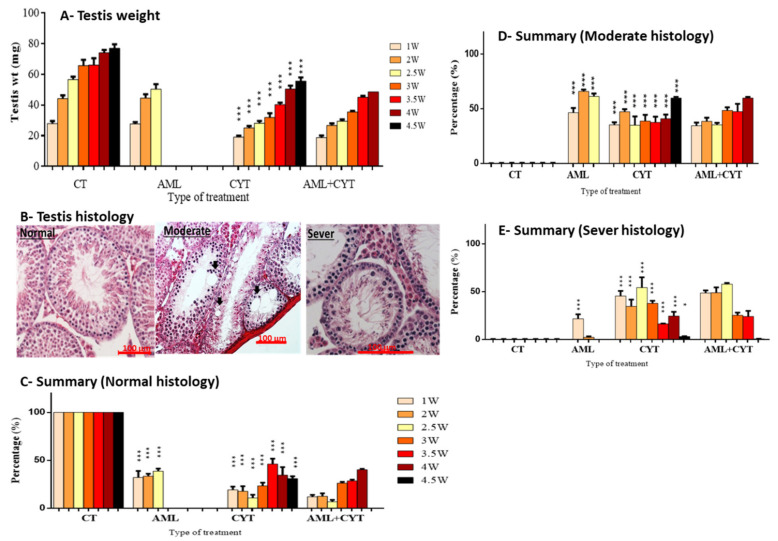
Effect of AML and CYT on testes weight and histology of immature treated mice. Mice were treated as described in Figure 1. Testes weights (mg) were measured at several time points post injection (1–4.5 week) (**A**). Histological sections were isolated from all treated groups at several time points post injection (1–4.5 week). The sections were classified according to severity level into three classes (**B**): percentage of normal seminiferous tubules (**C**), moderate (**D**) and severe (**E**). N = 4; n = 2–12. * (*p* < 0.05), *** (*p* < 0.005), @ (*p* < 0.05), @@ (*p* < 0.01) significant relative to AML group.

We weighed the body and testes of all mice involved in the study. We found that none of the treatments affected the body weight of the treated mice compared to the control group (data not presented). Additionally, injection of AML did not significantly affect testicular weight compared to control (Figure 2A). However, injection of CYT alone significantly reduced testicular weight during the 4.5 weeks post injection compared to control (Figure 2A). However, injection of CYT+AML did not significantly affect the testicular weight compared to injection with CYT alone (Figure 2A).

Sections from testes collected from all groups at several time points, depending on the survival of each treated group (CT and CYT, up to 4.5 weeks; AML, up to 2.5 weeks; and AML+CYT, up to 4 weeks post treatment), were stained with hematoxylin and eosin for histological analyses. They were analyzed according to the classification scale of normal (without damage of the tubules), moderate damage of tubules (fewer layers of cells, reduction in the size of tubules and presence of vacuoles) and severe damage of the tubules (single cell layer, very small tubules, increase in the diameter of the lumen and many more vacuoles), as shown in (Figure 2B). The quantification results of this classification are summarized in Figure 2C–E. 

Our results showed a significant decrease in the percentages of normal histology of the seminiferous tubules (STs) at all examined time points post treatment in the AML- (1–2.5 weeks), CYT- (1–4.5 weeks) and AML+CYT (1–4 weeks)-treated groups compared to control (Figure 2C). Additionally, we observed a significant increase in the percentages of moderate and severe histology of STs at all examined time points post treatment in the AML- (1–2.5 weeks), CYT- (1–4.5 weeks) and AML+CYT (1–4 weeks)-treated groups compared to control (Figure 2D,E). Testicular histology from testes of the AML+CYT-treated group showed similar percentages of damage of the seminiferous tubules as those of the CYT-treated group, with no additive/synergistic effect at any of the examined time points (1–4.5 weeks) (Figure 2D,E).

### 2.4. Effect of AML and CYT on Apoptosis of Spermatogenic Cells in Testicular Tissue of Immature Treated Mice

**Figure 3:**
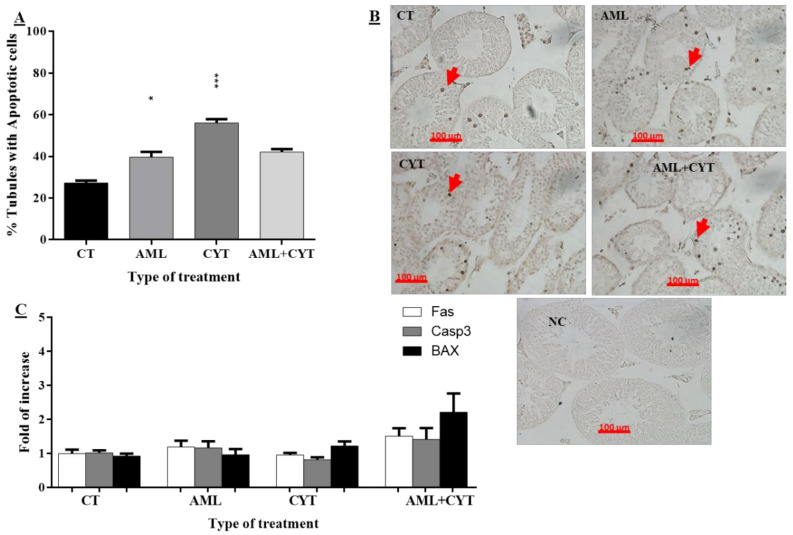
Effect of AML and CYT on apoptosis of spermatogenic cells in testicular tissue of immature treated mice. Mice were treated as described in Figure 1. Testes were removed after 2 weeks and fixed or used for RNA extraction. Fixed testicular tissues were examined by TUNEL assay to identify apoptotic cells. Apoptosis is defined as tubules with apoptotic cells and presented as the % of tubules with apoptotic cells. TUNEL assay (**A**) and quantification of staining (**B**). The RNA expression levels of Fas, Casp3 and BAX in the testes isolated from each treatment group were examined by qPCR analysis using specific primers, and housekeeping gene (GAPDH) was used as internal control. The results are presented as fold of increase compared to GAPDH (**C**). N (number of repeats of all treatments) = 3; n (number of mice examined from all the experiments for each treatment (TUNEL assay) = 6–7/group; #tubules/group = 40–100; n = 5–7/group (RNA expression). * (*p* < 0.05), *** (*p* < 0.001) significant relative to control group.

Histological analysis showed a clear impairment effect of AML and CYT on the cellular composition/structure of the seminiferous tubules. Therefore, we examined the effect of all treatments on the apoptosis process and the proliferation of cells in the seminiferous tubules of the treated groups. Testicular sections from all treated groups were examined by TUNEL assay to identify apoptotic cells in the seminiferous tubules (Figure 3B). For quantitative analysis, we considered/counted only tubules with more than three stained cells (this number of cells is present under normal conditions) as tubules positive for apoptosis. Our results showed a significant increase in the percentage of tubules with positive apoptotic cells in the AML group and the CYT group 2 weeks post treatment compared to the control group (Figure 3A). However, the percentage of apoptotic seminiferous tubules in the AML+CYT group was similar to that of the AML- and CYT-treated groups (Figure 3A). On the other hand, our results showed that the RNA expression levels of intrinsic and extrinsic signals of apoptosis, such as Fas, casp3 and bax, in the testicular tissue of immature mice treated with AML or CYT (alone) or in combination (AML+CYT) were similar to those of the control group (Figure 3C). Additionally, the expression levels of Fas, casp3 and bax did not show a significant change in the AML+CYT group compared to the AML and CYT groups (Figure 3C).

These results may suggest that AML and CYT treatments increase the percentage of tubules with positive apoptotic stained cells. It is known that CYT has the ability, like other cytotoxic medications, to induce damage in the proliferating cells, which leads to an increase in the percentage of apoptotic cells.

### 2.5. Effect of AML and CYT on the Expression Levels and Number of Spermatogenic Cells of Immature Treated Mice

**Figure 4:**
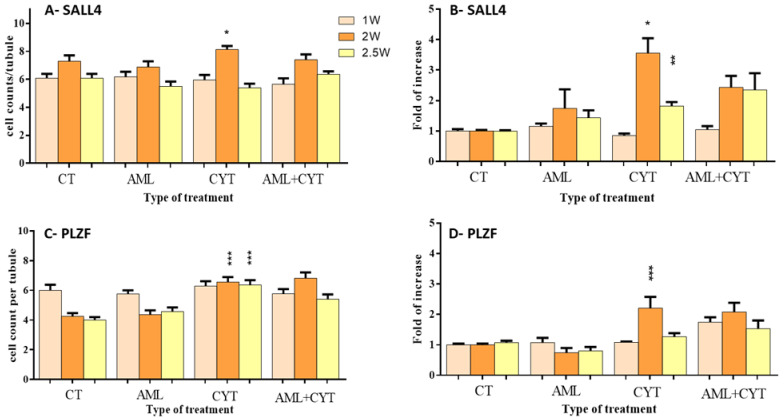
Effect of AML and CYT on the presence and expression levels of premeiotic markers. Mice were treated as described in Figure 1. Testes were removed after 1, 2 or 2.5 weeks and fixed or used for RNA extraction. Fixed testicular tissues were immunoassayed with specific anti-SALL4 and anti-PLZF antibodies to identify the premeiotic cells. SALL4-positive stained cells are presented as SALL4/tubule (**A**). The RNA expression levels of SALL4 in the testes isolated from each treatment group were examined by qPCR analysis using specific primers (**B**). PLZF-positive stained cells are presented as PLZF/tubule (**C**). The RNA expression levels of PLZF in the testes isolated from each treatment group were examined by qPCR analysis using specific primers (**D**), and housekeeping gene (GAPDH) was used as internal control. The results are presented as fold of increase compared to GAPDH (**B**). N = 3; n = 7–8/group; #tubules/group = 40–100; n = 5–7/group (RNA expression). * (*p* < 0.05), ** (*p* < 0.01), *** (*p* < 0.001) significant relative to control group.

Following the examination of the effect of AML and CYT on the apoptosis process in testes, we investigated the effect of those treatments on cells of the seminiferous tubules at different stages of spermatogenesis, such as SALL4 and PLZF (premeiotic stage), CREM (meiotic) and acrosin (meiotic/post-meiotic). Following immunohistochemical staining (IHC) for SALL4 (see Figure S1A) and PLZF (see Figure S1B), we counted the number of positively stained cells/tubule and summarized the results. In addition, we examined the effect of AML and CYT on the expression levels of SALL4 and PLZF in the testes of the treated mice. The data show that AML and AML+CYT treatments did not significantly affect the number of SALL4-stained cells/tubule, PLZF-stained cells/tubule or their expression levels at any of the examined time points compared to the control (Figure 4A–D). However, CYT treatment significantly increased the number of SALL4-stained cells/tubule 2 weeks post treatment and the expression levels 2 and 2.5 weeks post treatment compared to the control group (Figure 4A). It should be noted that the expression of SALL4 2.5 weeks post treatment was lower than at 2 weeks post treatment (Figure 4B). On the other hand, CYT treatment significantly increased the number of PLZF-stained cells/tubule 2 and 2.5weeks post treatment and the expression levels 2 weeks post treatment compared to the control group (Figure 4C,D).

**Figure 5:**
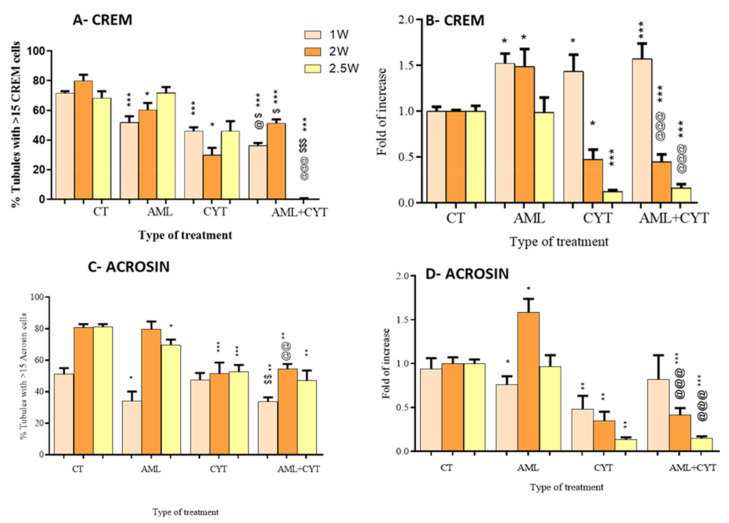
Effect of AML and CYT on the presence and expression levels of meiotic/post-meiotic markers. Mice and testes were treated as described in Figure 1. Testes were removed after 1, 2 or 2.5 weeks and fixed or used for RNA extraction. Fixed testicular tissues were immunoassayed with specific anti-CREM and anti-acrosin antibodies to identify meiotic/post-meiotic cells. Tubules with more than 15 CREM-positive cells (**A**) and ACROSIN-positive cells (**C**) are presented as % of tubules. The RNA expression levels of CREM (**B**) and ACROSIN (**D**) in the testes isolated from each treatment group were examined by qPCR analysis using specific primers, and housekeeping gene (GAPDH) was used as internal control. The results are presented as fold of increase compared to GAPDH (**B**). N (number of repeats of all treatments) = 3; n = (number of mice examined from all the experiments for each treatment (immunostaining) = 6–9/group; #tubules/group = 40–120; n = 5–7/group (RNA expression). * (*p* < 0.05), ** (*p* < 0.01), *** (*p* < 0.001) significant relative to control group. @ (*p* < 0.05), @@ (*p* < 0.01), @@@ (*p* < 0.001) significant relative to AML group. $ (*p* < 0.05), $$ (*p* < 0.01), $$$ (p<0.001) significant relative to CYT group.

Next, we investigated the effect of AML and CYT alone or in combination on the meiotic (CREM) and post-meiotic (ACROSIN) stages. The quantification analysis of immunofluorescent staining and expression levels of CREM and ACROSIN was performed (Figure 5A–D). For quantitative analysis, we considered/counted only tubules with more than 15 CREM-stained cells (see Figure S2A). Our results show a significant decrease in the percentage of tubules with more than 15 CREM-stained cells in the AML group (1–2 weeks post treatment) and CYT alone or in combination with AML (1–2.5 weeks post treatment) compared to the control group (Figure 5A), whereas the percentages of tubules with more than 15 CREM-stained cells were significantly decreased 1–2.5 weeks post treatment in the AML+CYT group compared to AML alone and CYT alone (Figure 5A). However, the RNA expression levels of CREM were significantly increased 1–2 weeks post AML treatment compared to control (Figure 5B). Furthermore, a significantly increased was detected 1 week post CYT treatment, but a significant decrease was detected 2–2.5 weeks post CYT treatment compared to control (Figure 5B). On the other hand, treatment of immature mice with AML+CYT did not significantly affect CREM expression levels compared to treatment with CYT alone (Figure 5B). 

In parallel, the analysis of the meiotic/post-meiotic stage was performed by evaluating the immunofluorescent staining of ACROSIN in the same AML and CYT immature treated mice (see Figure S2B). The tubules included in the analysis of the post-meiotic stage were those with more than 15 ACROSIN-positive cells. Our results show that AML and CYT significantly decreased the percentage of tubules with more than 15 ACROSIN-positive cells 1 and 2.5 weeks post AML and 2–2.5 weeks post-CYT treatment compared to the control group (Figure 5C). On the other hand, AML+CYT significantly decreased the percentage of tubules with more than 15 ACROSIN-positive cells in the first week compared to CYT but was similar to CYT 2 and 2.5 weeks post treatment (Figure 5C). 

RNA expression results showed that AML significantly decreased the expression levels of ACROSIN in testicular homogenates of immature treated mice in the first week post treatment but increased its expression in the second week and was similar 2.5 weeks post treatments compared to control (Figure 5D). However, CYT significantly decreased the expression levels of acrosin in all the examined periods compared to the control group (Figure 5D). Treatment with AML+CYT did not significantly affect the expression levels of ACROSIN compared to CYT treatment alone (Figure 5D). 

Our results show that AML, CYT and AML+CYT significantly decreased the meiotic and post-meiotic cells at several points post treatment compared to control. Furthermore, the AML+CYT-treated group showed a similar effect to that of the CYT only group on meiotic and post-meiotic cells at almost all examined time points (Figure 5A–D).

### 2.6. Effect of AML and CYT on Cell Proliferation of Spermatogenic Cells in Testicular Tissue of Immature Treated Mice

**Figure 6:**
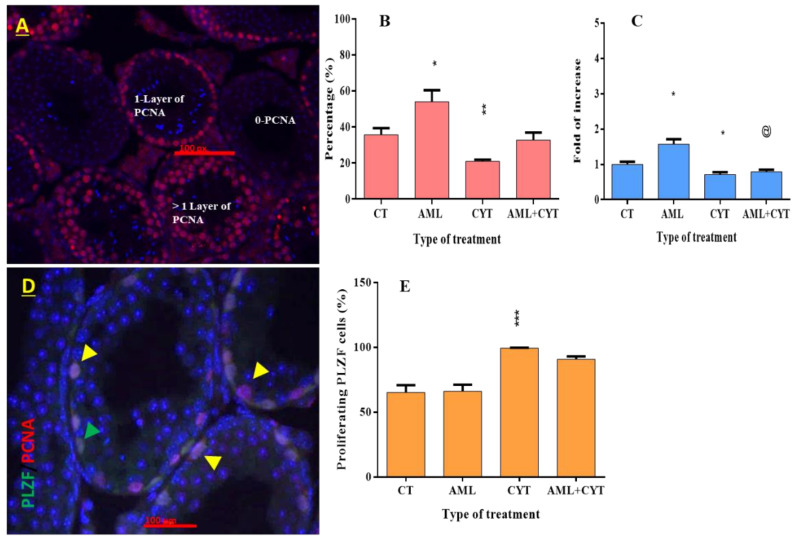
Effect of AML and CYT on testicular cell proliferation and cell proliferation of premeiotic cells in testicular tissue of immature treated mice. Mice and testes were treated as described in Figure 1. Fixed testicular tissues were immunoassayed with specific anti-PCNA antibodies to identify the proliferating cells and anti-PLZF antibodies to identify the premeiotic cells. Tubules with PCNA-positive cells are presented as % of tubules with one layer of PCNA-positive cells. Immunostaining (**A**) and quantification of the staining (**B**). The RNA expression levels of PCNA in the testes isolated from each treatment group were examined by qPCR analysis using specific primers (**C**), and housekeeping gene (GAPDH) was used as internal control. The results are presented as fold of increase compared to GAPDH. Testicular tissues were immunoassayed with specific anti-PLZF and anti-PCNA antibodies to identify the proliferating premeiotic cells. Tubules with doubled positive cells are presented as % of proliferating PLZF cells. Immunostaining (**D**) and quantification of the staining (**E**). N = 3; n = 5–7/group; #tubules/group = 40–110; n = 5–7/group (RNA expression). * (*p* < 0.05), ** (*p* < 0.01), *** (*p* < 0.001) significant relative to control group. @ (*p* < 0.05) significant relative to AML group. Green arrow indicates PLZF-positive stained cells. Yellow arrow indicates proliferating PLZF-positive stained cells.

Testicular sections from all treatment groups were examined for the presence of PCNA-positive cells (PCNA is a proliferation cell marker) by immunofluorescent staining to identify the localization of proliferating cells in the seminiferous tubules of testes from mice 2 weeks post treatment (the last period that mice survived post AML treatment) (Figure 6). Positive PCNA-labeled cells were identified near the basement of the seminiferous tubules (Figure 6A). The tubules included in our results were those with a single layer of PCNA-positive staining only (one layer of PCNA) (this layer of cells contains mainly spermatogonial cells). Quantification analysis of immunofluorescent staining showed that the percentage of tubules with one layer of PCNA-positive staining significantly increased in the AML group 2 weeks post treatment compared to control; CT (Figure 6B), whereas a significant decrease in the percentage of tubules with one layer of PCNA-positive staining was detected in the CYT-treated group 2 weeks post treatment compared to control; CT (Figure 6B). The percentages of seminiferous tubules with one layer of PCNA-positive staining from testes of the AML+CYT -treated group showed a significant decrease compared to AML treatment alone and a significant increase compared to CYT treatment alone (Figure 6B). The RNA expression levels of PCNA in the testicular tissue of immature mice treated with AML or CYT alone or in combination were similar to the results of PCNA immunostaining (Figure 6C).

Thus, our results show that CYT treatment significantly decreased proliferative cells 2 weeks post treatment, whereas AML significantly increased proliferative compared to control.

In order to identify the proliferating cells counted in previous immunostaining assays, we conducted double immunostaining using specific antibodies against (PCNA) and spermatogonial stem cell marker (PLZF) (Figure 6D). Our results showed that the CYT treatment significantly increased the percentage of cells double-stained with PCNA and PLZF relative to the untreated control group (Figure 6E). AML treatment had no effect on those cells, and there was no additive or synergistic effect of AML+CYT on those cells. These results suggest that CYT, but not AML, induces the proliferation of SSCs located in the basement membrane of tubules.

### 2.7. Effect of CYT and AML+CYT on Sperm Concentration

To examine the effect of CYT and CYT+AML on the generation of sperm, we sacrificed the mice from all treated groups at the last possible time point of their survival (4 weeks post treatment) and isolated sperm from their epididymis. Our results show the complete absence of sperm from CYT- and CYT+AML-treated groups compared to the control group (CT), which showed normal generation of sperm at a concentration of (1.07 ± 0.13) × 10^6^/mL. 

### 2.8. Effect of AML and CYT on the Levels of Testicular Growth Factors, Proinflammatory and Anti-Inflammatory Cytokines of Immature Treated Mice

**Figure 7:**
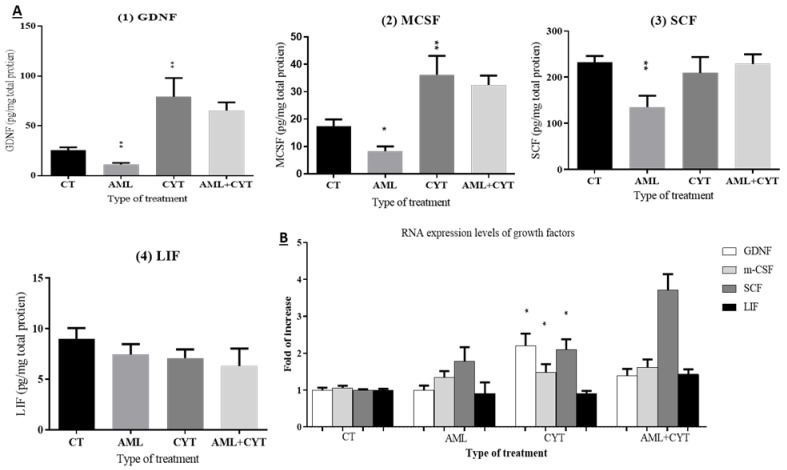
Effect of AML and CYT on the levels of testicular growth factors of immature treated mice. Mice and testes were treated as described in Figure 1. Testes were removed after 2 weeks and used for protein homogenization or used for RNA extraction. Protein homogenates were used to measure growth factor protein levels with specific ELISA kits (**A**). The RNA expression levels of growth factors and cytokines in the testes isolated from each treatment group were examined by qPCR analysis using specific primers (**B**), and housekeeping gene (GAPDH) was used as internal control. The results are presented as fold of increase compared to GAPDH. N = 4; n = 5–7/group (protein levels); n = 5–7/group (RNA expression). * (*p* < 0.05), ** (*p* < 0.01) significant relative to control group.

Following the examination of the effect of AML and CYT on spermatogenesis, we investigated the effect of those treatments on the testicular growth factors and cytokines mainly secreted from testicular somatic cells (microenvironment of the SSCs) involved in the regulation of spermatogonial cell maintenance, proliferation and differentiation. Our results show that AML significantly decreased the protein levels of GDNF, MCSF and SCF but not LIF compared to control (Figure 7A; 1–4, respectively). However, CYT significantly increased the protein levels of GDNF and MCSF, although it did not affect the protein levels of SCF and LIF at the same time point post injection. In terms of RNA expression level, AML did not show a significant effect on any of the examined growth factors, and CYT showed a similar effect on the expression levels and protein levels of the examined factors (Figure 7A,B).

**Figure 8:**
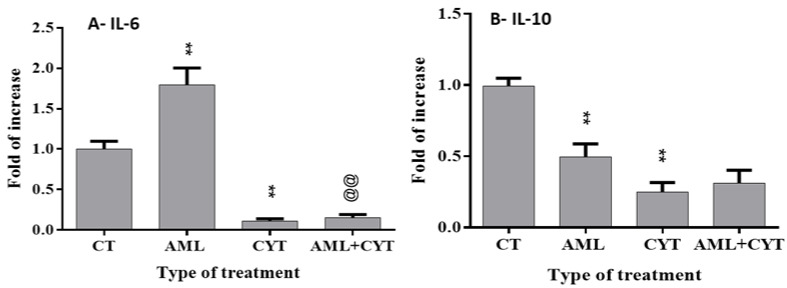
Effect of AML and CYT on the levels of testicular proinflammatory and anti-inflammatory cytokines of immature treated mice. Mice and testes were treated as described in Figure 1. Testes were removed after 2 weeks and used for RNA extraction. The RNA expression levels of IL-6 (**A**) and IL-10 (**B**) in the testes isolated from each treatment group were examined by qPCR analysis using specific primers, and housekeeping gene (GAPDH) was used as internal control. The results are presented as fold of increase compared to GAPDH. N = 4; n = 5–7/group (RNA expression). ** (*p* < 0.01) significant relative to control group.@@ (*p* < 0.01) significant relative to AML group.

In addition, our results show that AML treatment significantly increased the RNA expression level of the proinflammatory cytokine (IL-6), whereas CYT treatment decreased it significantly (Figure 8A). Furthermore, AML+CYT did not show an additive or synergistic effect on IL-6 expression levels (Figure 8A). However, AML alone and CYT alone significantly decreased the RNA expression level of the anti-inflammatory cytokine (IL-10) (Figure 8B). On the other hand, AML+CYT did not show an additive or synergistic effect on IL-10 expression levels (Figure 8B).

## 3. Discussion

In this study, we demonstrated that the AML disease model in sexually immature mice is successfully developed. There was an increase in monocytes and granulocytes in the AML-cell-injected mice, which is consistent with the analysis used to diagnose pediatric AML in humans [33]. In addition, AML cells were demonstrated in the blood vessels of the testes, which approved the translocation of AML cells from the peritoneum, reaching the bloodstream, including the testicular blood vessels. Histological analysis showed for the first time that AML alone affected the development of spermatogenesis at the adult age in AML-treated immature mice. The damage is clear in the structure/histology of the seminiferous tubules (including the appearance of vacuoles) and in the increasing apoptotic cells in the seminiferous tubules. Our results showed that cells of the meiotic/post-meiotic stages of spermatogenesis are the main cells affected by AML. This could be related to the imbalance in testicular growth factors (SCF, GDNF and MCSF) that are important in spermatogonial development (providing growth and/or differentiation factors and a normal niche), thus reducing differentiation and development of the meiotic and post-meiotic cells. In parallel, AML increased the proliferation of cells in the basement membrane of the seminiferous tubules (as examined by PCNA), alongside the percentage of tubules with apoptotic cells (TUNEL assay). These results are in harmony with a recent study that showed that AML disease induced histological damage and decreased the count of meiotic/post-meiotic cells [26]. In addition, AML disease increased the percentage of seminiferous tubules with apoptotic cells in adult mice [8]. Additional studies focused on the effect of cancers, including AML, in adult male fertility; these studies showed that patients with cancer disease had impairment in their semen parameters even before anticancer treatment [4,5,7,34]

AML increased the RNA expression levels of testicular IL-6 (a proinflammatory cytokine), which is secreted by testicular somatic and spermatogenic cells and involved in the regulation of spermatogenesis under normal conditions and in response to inflammatory stimuli [34,35,36,37]. This suggests that AML may induce inflammatory processes in the testis and/or lead to an imbalance in testicular cytokines that are involved, in a direct or indirect manner, in the environment/niche of the developed germ cells and thus may impair normal spermatogenesis. This result is in harmony with previous studies that showed that the RNA expression levels and/or protein levels of IL-6 increased following systemic inflammation and in testicular cancer. It was suggested that IL-6 may participate and promote different pathophysiologies, such as testicular cancer, inflammation and infertility [26,37,38,39,40,41]. In contrast to IL-6, AML decreased the RNA expression levels of testicular IL-10 (anti-inflammatory cytokine), which is produced by somatic cells, mast cells and macrophages in the testicular tissue and is involved in the regulation of inflammatory disorders in the testes [34,35]. Our result may suggest that AML disrupts the immune-tolerant status in the microenvironment of the testes that is maintained by IL10. These results are in correlation with previous studies that showed that IL-10 levels remained more stable than those of IL-6 in normal conditions or under testicular cancer [8,26,34,38,39,40,42].

In parallel, we demonstrated that CYT decreases the testicular weight in adult-age immature treated mice. Our results are in harmony with previous studies that showed that cytotoxic agents such CYT and busulfan decreased the weight of developing organs, such as testes and placenta, in rodents [20,43,44]. Histological analysis showed that CYT induced damage in testes, which was further confirmed by the increase in cell apoptosis and the decrease in the proliferation of cells in the seminiferous tubules. This could be explained by the ability of CYT and other chemotherapies to target highly proliferating cells. These results are in harmony with previous studies that showed CYT impairs spermatogenesis, increases the percentage of tubules with apoptotic cells and decreases sperm parameters in adult mice [7,8,20]. 

CYT alone and AML alone or in combination (AML+CYT) significantly increased the apoptosis of germ cells in the seminiferous tubules, as examined by TUNEL assay. However, the RNA expression levels of the examined intrinsic and extrinsic apoptotic factors (FAS, BAX and Casp3) in the testicular tissues were not affected by any of the treatments. The discrepancy between the results of TUNEL assay and those of RNA expression could be related to the counting of cells in the seminiferous tubules that were stained positively in the TUNEL assay, whereas the RNA analyses were performed on testicular tissue. An additional option is the possibility of differences in the regulatory processes (transcription/translation and RNA stability) between RNA expression and the final process of apoptosis (dead cells). 

Furthermore, CYT increased the count of SSCs (SALL4 and PLZF cells), and testicular growth factors, including GDNF. We suggest that CYT increased GDNF within the niche of the spermatogonial cells, which had a critical role in SSC self-renewal and maintenance by binding to its receptors, GFRa1, that were mostly expressed in undifferentiated spermatogonia and RET. This regulation could involve RET phosphorylation that induces SRC family and PI3K/AKT activation, allowing for the proliferation of SSCs [45,46,47]. This is in agreement with a previous study that showed that GDNF and GFRa1 were involved in SSCs self-renewal ability in mice [48]. Another study reported that busulfan promotes the protection of SSCs by regulating the expression of GDNF [49]. However, CYT decreases meiotic/post-meiotic (CREM and ACROSIN) cells and sperm concentration. We suggest that CYT targets highly proliferating cells, which imbalances the niche, leading to a decrease in cells of the meiotic/post-meiotic stages. Our results align with previous studies that showed a similar effect of CYT on meiotic/post-meiotic cells and sperm concentration in adult mice [7,8].

Our results show that the effect of AML and CYT together (AML+CYT) showed a similar effect to that of CYT in most of the examined parameters and factors, except SCF and percentage of apoptotic cells. This may reflect the severe effect of CYT on spermatogenesis and that the mechanism of CYT is major factor. However, this mechanism is different in some functions of the spermatogenesis (cellular apoptosis and expression of SCF). 

In conclusion, our results show that AML alone, in the prepubertal age, affects male fertility in the adult age. In addition, treatment of boys with AML with CYT could adversely affect their fertility at adult age.

## 4. Materials and Methods

### 4.1. Mice

Two-week-old C57Bl/6 mice were used in the present study. The mice were purchased from Harlen Laboratories Ltd., Jerusalem, Israel. They were appropriately handled at the animal house of the Faculty of Health Science at Ben-Gurion University in Negev, Israel, according to the established handling protocols. The mice were sacrificed at several time points post injection, blood was collected from the heart, and the body and testes were weighed. 

This study was performed in accordance with the Guiding Principles for the Care and Use of Research Animals promulgated by the Society for the Study of Reproduction and was confirmed by the Ben-Gurion University Ethics Committee for Animal Use in Research (IL-70-11-2016). 

### 4.2. Development of a Model of AML in Immature Mice

Mouse AML cell line [murine C1498 (TIB-49) purchased from American Type Culture Collection (Rockville, MD, USA)] was used. We intraperitoneally (i.p) injected 3 × 10^4^ cells/100µL PBS according to the protocol of the murine C1498 Cell Line used in adult mice [7,50,51]. Before use, the cells were cultured in RPMI 1640 medium supplemented with 10% FBS, penicillin (100 U/mL), streptomycin (0.1 mg/mL) and 10 mM HEPES (pH = 7.4) in a humidified atmosphere of 95% air and 5% CO_2_ at 37 °C. Cytarabine powder was purchased from SIGMA (Sigma-Aldrich Israel Ltd., Rehovot, Israel). The powder was dissolved in sterile PBS. CYT (140 mg/kg) was injected intraperitoneally (i.p) into each mouse. The injections were performed every 12 h, three times 24 h after C1498 cell injection. This was performed according to the protocol described by Cano et al. and our previous study that used adult mice [7,52]. As a control, mice were injected with 100 µL of sterile PBS.

### 4.3. Hematological Analysis

Blood samples were collected from the heart of the mice immediately after CO_2_ sacrifice. Monocyte and granulocyte levels were measured by an Exigo H400 veterinary hematology analyzer according to the manufacturer instructions. Giemsa staining was used to morphologically identify AML cells in blood samples.

### 4.4. Immunofluorescent Staining of Testicular Tissues

Immunofluorescent staining was performed as described previously [53]. Testicular tissues were fixed in Bouin’s solution (Kaltek, Pavoda, Italy) and paraffin-embedded. Sections of 5 µm were placed on Superfrost Plus slides (Thermo, Braunschweig, Germany) for histological evaluation and immunofluorescence staining of CREM (Polyclonal rabbit anti-mouse, 1:200; Proteintech Croup, Inc; Rosemont, IL, USA Cat. No. 12131-1AP), ACROSIN (Polyclonal rabbit anti-mouse, 1:1000; Novus Biologicals, LLC, Centennial, CO USA, Cat. No. A113694), PCNA (Monoclonal mouse anti-mouse, 1:100, Santa Cruz Biotechnology, Santa Cruz, CA, USA Cat. No.) and α-smooth (Polyclonal goat anti-mouse, 1:200; Abcam, Cambridge, UK Cat. No. ab21027). The specificity of the staining was also examined in the testicular tissue using the relevant IgG isotype as the negative control. Secondary antibodies used in this study were: donkey anti-rabbit IgG (Cy3) (Jackson immunoresearch, West Grove, PA, USA; 1:700), donkey anti-goat IgG (Cy3) (Jackson immunoresearch, 1:700), goat anti-mouse IgG (Cy3) (Jackson immunoresearch, 1:500) and goat anti-rabbit IgG (Alexa Fluor 488) (Jackson immunoresearch, 1:200). The slides were examined for staining using a fluorescence microscope (Nikon Eclipse 50 I; Tokyo, Japan).

### 4.5. Immunohistochemistry Staining of Testicular Tissues

Immunohistochemistry staining was performed as described in the protocol (ImmPRESS REAGENT, Anti-Rabbit IgG, Cat. No. MP-7401). Briefly, the slides were prepared exactly as mentioned above. Then, the slides were deparaffinated and dehydrated by xylene and ethanol, respectively (BIO-LAB, Jerusalem, Israel). After washing with a phosphate-buffered solution (PBS) (Biological Industries, Beit HaEemek, Israel), antigen retrieval of the sections was performed in heated sodium citrate solution (Sigma, St. Louis, MO, USA) in a warm microwave for 7 min (twice). After washing, the nonspecific adhesion sites in the tissues and cells were blocked for 90 min at room temperature using a horse serum. Following the removal of the blocking buffer, the first antibodies were added as follows: PLZF (Polyclonal rabbit anti-mouse, 1:100, Santa Cruz Biotechnology, Santa Cruz, CA, USA, Cat. No. sc-22839), SALL4 (Polyclonal rabbit anti-mouse, 1:200; Abcam, Cambridge, UK, Cat. No. ab29112). After overnight incubation at 4 °C, the slides were washed, and the specific secondary antibodies were added compatibly to the first antibodies for 60 min at room temperature. After washing, the slides were dried. The specificity of the staining was also examined in the testicular tissue using the relevant IgG isotype as the negative control. The slides were examined for staining using a fluorescence microscope (Nikon Eclipse 50 I; Tokyo, Japan).

### 4.6. Hematoxylin Eosin Staining

This staining was performed as described previously [53]. Briefly, the tissues were deparaffinized in a K-clear solution, rehydrated in different ethanol concentrations and, later on, in deionized water. The slides were incubated in a hematoxylin reagent, then rinsed in deionized water and rinsed in tap water to allow for development of the staining. Next, the slides were dipped in an eosin solution, followed by DDW (double-distilled water). This was followed by dehydration by rinsing the slides in ascending ethanol concentrations and rinsing in K-clear. The slides were dried and coverslips using permount.

### 4.7. Real-Time Quantitative PCR

RNA was extracted according to the company protocol (Sigma (GenElute Mannalian Total RNA Miniprep)) and according to our previous publication [54]. cDNA synthesis was performed according to the qScript cDNA Synthesis Kit (Quantabio, Beverly, MA, USA) using random hexamers, and qPCR was performed using specific primers for each examined marker: GAPDH Fw-5-5′ACCACAGTCCATGCCATCAC-3′,Rv-5′-CACCACCCTGTTGCTGTAGCC-3′. PLZF Fw-5′-AGCTTGAAATACGTGGCCAGA-3′, Rw-5′-TGAGCAGTTCACACTTCATCCC-3′. SALL4 Fw-5′-GAAAGCCCACAATTTCTCCTG, Rv-5′- AGGAAACAGGCAGTTTTCCAA-3′. CREM Fw-5′-TTCTTTCACGAAGACCCTCA-3′, Rw-5′-TGTTAGGTGGTGTCCTTCT-3′. ACROSIN Fw-5′-TGTCCGTGGTTGCCAAGGATAACA-3′, Rv-5′-AATCCGGGTACCTGCTTGTGAGTT-3′. PCNA Fw-5′-ATGATCTTGACGCTAAATGCAG-3′, Rw-5′-TGGAAGAGAGAAAAGCCCA-3′Fas Fw-5′-GCTGGCTCACAGTTAAGAGTT-3′, Rv-5′-GTTGGTGTACCCCCATTCATT-3′. Bax-Fw-5′-ACCAGCTCTGAACAATCATG-3′, Rv-5′-TGGATCCAGACAAC. Casp3 Fw-5′-AGCTGTACGCGCACAGCTA-3′, Rv-5′-CCGTTGCCACCTTCTGTTA-3′. MCSF w-5’-CCCATATTGCGACACCGAA-3′, Rw-5’-AAGCAGTAACTGAGCAACGGG-3′. SCF Fw-5’-TGAGCCCTTATGCCACACAAT-3′, Rw-5’-AAGATGATCCCAAACGCTCGT-3′. GDNF Fw-5’-GCCCCTGCTTTCTATCTGCT-3′, Rw-5’-AGCCTTCTGAATGCGTGGTT-3′. LIF Fw-5’-TAGCGGCTTCAGAAGGGAAAT-3′, Rw-5’-AAGGAAAGGAAAGAGGGAGAGC-3′. IL-1α Fw-5’-GAAGCTCGTCAGGCAGAAGT-3′, Rw-5’-GTGCACCCGACTTTGTTCTT-3′. IL-10 Fw-5′- CGGGAAGACAATAACTGCACCC-3′, Rv-5′- CGGTTAGCAGTATGTTGTCCAGC-3′. IL-6 Fw-5′-GACGATACCACTCCCAACAGACC-3′, Rv-5′- ATGCTTAGGCATAACGCACTAGGTT-3′.

The qPCR reaction was performed following the method described our previous study [53]. The “threshold cycle” (C*t*) values were defined, representing the cycle number at which the sample fluorescence rises statistically above the background and crossing points (CP) for each transcript. The relative quantity of gene expression was analyzed using the 2^−ΔΔ^*^C^^t^* method. The results are expressed as the fold of increase related to the GAPDH of the same examined sample.

### 4.8. TUNEL Immunohistochemistry

Immunohistochemical apoptotic detection was carried out using a commercial kit (DeadEnd™ Fluorometric TUNEL System, Promega, Madison, WI, USA). The TUNEL assay was performed according to the manufacturer’s instructions. The system directly detects apoptotic cells in situ at the single-cell level in paraffin-imbedded tissue. The positive staining was examined using a light microscope. 

### 4.9. Sperm Extraction and Sperm Concentration Measurement

Mice were sacrificed four weeks post treatment, and sperm was extracted according to the method described in our previous study [7].

### 4.10. Enzyme-Linked Immunosorbent Assay (ELISA)

The protein levels of growth factors (GDNF, MCSF, LIF and SCF) in testicular homogenates were evaluated by specific Duoset ELISA for each examined growth factor. The following kits were used. according to the manufacturers’ instructions: GDNF (DY212; R&D systems Inc., Minneapolis, MN, USA), MCSF (DY416; R&D systems Inc., Minneapolis, MN, USA), LIF (DY449; R&D Systems Inc., Minneapolis, MN, USA) and SCF (DY455; R&D systems Inc., Minneapolis, MN, USA). Briefly, 96-well plates were separately coated with 100 µL capture antibodies against each growth factor overnight at room temperature, washing 3 times with wash buffer (0.05% Tween20 in PBS, pH 7.2–7.4), followed by blocking with 300 µL of BSA 1% at room temperature for at least 1 h. The washing step was repeated, as mentioned previously. Samples and the standards were then added and incubated at room temperature for 2 h. After the washing step, 100 µL of detection antibodies, anti-GDNF, anti-MCSF or anti-LIF and anti-SCF, were added and incubated at room temperature for 2 h. The plates were then washed as mentioned before. A volume of 100 µL of the working dilution of streptavidin-HRP (R&D systems Inc., Minneapolis, MN, USA) was added and incubated for 20 min at room temperature, and another washing procedure was performed. A volume of 100 µL of substrate solution was added to each well, and the plate was incubated for 20 more minutes. At the end of the incubation, 50 µL of stop solution was added, and the optical density of each well was measured using a microplate reader set to 450 nm.

### 4.11. Evaluation of Total Protein in Testicular Homogenates

Protein assay dye reagent concentrate (Bio-Rad laboratories Inc., Hercules, CA, USA) was diluted in PBS (1:5) and filtered. Each well was covered with 200 µL of the diluted dye reagent. The protein standard, albumin bovine, was purchased from MP Biomedicals, Irvine, CA, USA. The standard protein was diluted in duplicates (1 mg/mL, 0.5 mg/mL, 0.25 mg/mL, 0.125 mg/mL, 0.0625 mg/mL). Each well was covered with 10 µL of standard protein/sample. The optical density of each well was then measured by a microplate reader set to 595 nm.

### 4.12. Statistical Analysis

The statistics values were calculated according to Av. ± SEM. The statistical significance was examined by two-tailed *t*-test and was shown as *p*-value: *^,@,$^ *p* < 0.05, **^,@@,$$^ *p*-value < 0.01, ***^,@@@,$$$^ *p*-value < 0.001.

## Data Availability

The data that support the findings of this study are available from the corresponding author upon reasonable request.

## Supplementary Materials

The following supporting information can be downloaded at: https://www.mdpi.com/article/10.3390/ijms23074013/s1.
