# Supplementary material for: Effect of Chemotherapy Cytarabine and Acute Myeloid Leukemia on the Development of Spermatogenesis at the Adult Age of Immature Treated Mice
Source: Int J Mol Sci. 2022 Apr 4;23(7):4013. doi: 10.3390/ijms23074013 (PMC8999636; doi:10.3390/ijms23074013)
Supplement: Supplementary file 1 [file ijms-23-04013-s001.zip › ijms-1630775-supplementary.pdf]

Supplementary

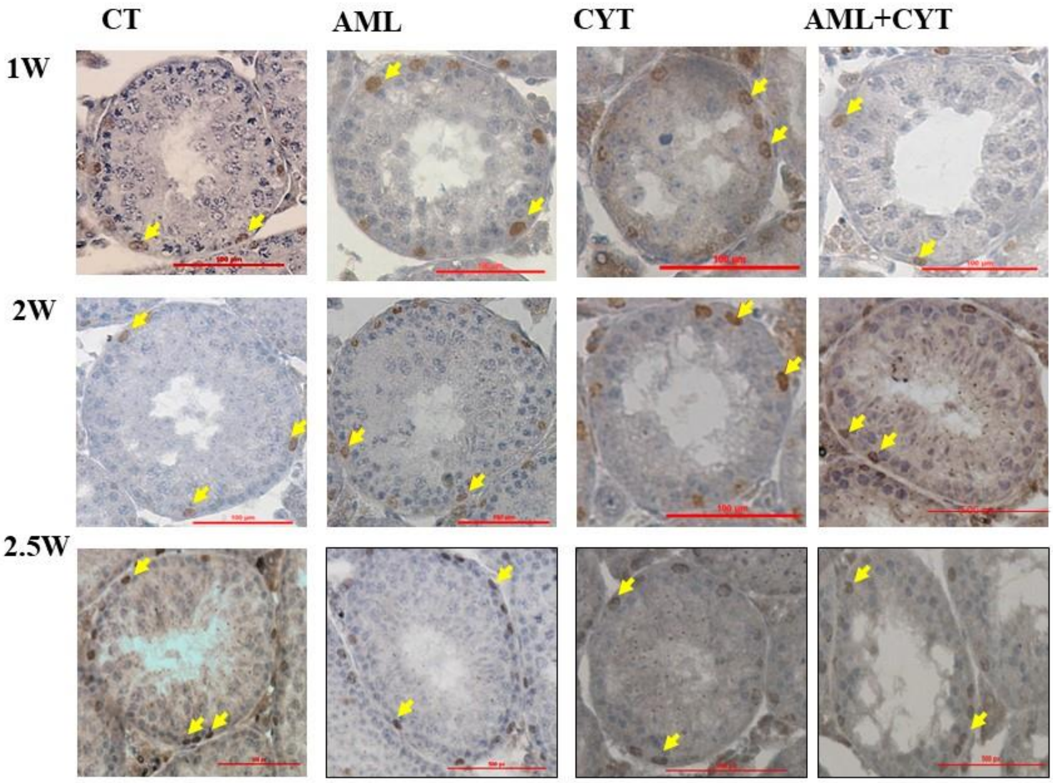

Figure S1A. Localization of SALL4-positive stained cells by immunohistochemistry staining in testicular sections from immature-treated mice after different treatment time points.

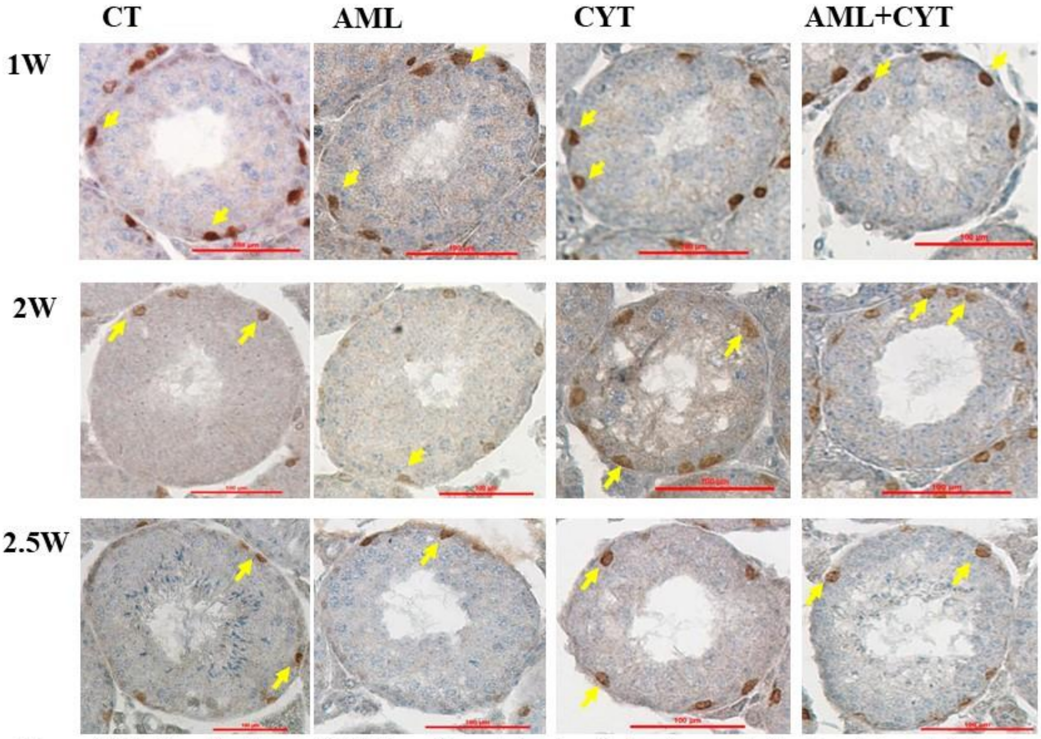

Figure S1B. Localization of PLZF-positive stained cells by immunohistochemistry staining in testicular sections from immature-treated mice after different treatment time points.

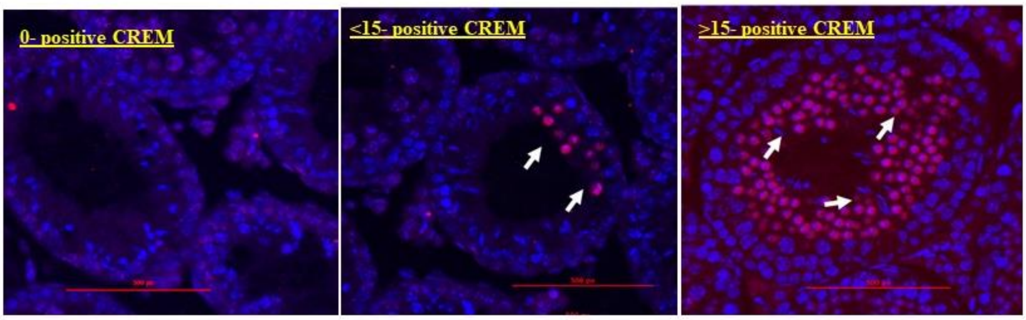

Figure S2A. Classification scale of tubules with CREM-positive stained cells in testicular sections from immature-treated mice.

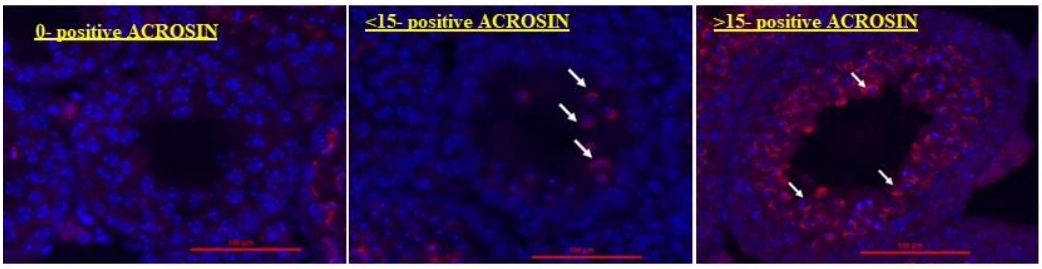

Figure S2B. Classification scale of tubules with ACROSIN-positive stained cells in testicular sections from immature-treated mice.
